# Expression of astrocyte elevated gene-1 closely correlates with the angiogenesis of gastric cancer

**DOI:** 10.3892/ol.2014.1950

**Published:** 2014-03-07

**Authors:** SHUHUA LI, XUEFENG GUO, XUDONG MA, CUILAN TANG, ZUNFU KE, WENHUA HUANG

**Affiliations:** 1 Department of Pathology, The First Affiliated Hospital of Sun Yat-Sen University, Guangzhou, Guangdong 510080, P.R. China; 2 Department of Gastrointestinal Surgery, The Sixth Affiliated Hospital of Sun Yat-Sen University, Guangzhou, Guangdong 510655, P.R. China; 3 Zhangzhou Affiliated Hospital of Fujian Medical University, Zhangzhou, Fujian 363000, P.R. China; 4 Department of Anatomy, School of Basic Medical Science, Southern Medical University, Guangzhou, Guangdong 510515, P.R. China

**Keywords:** astrocyte elevated gene-1, gastric cancer, angiogenesis, vascular endothelial growth factor, hypoxia-inducible factor-1α

## Abstract

Previous studies have demonstrated that astrocyte elevated gene-1 (AEG-1) is overexpressed in several cancer types and that its upregulation may promote cell proliferation, cell transformation and tumor progression. The present study investigated the expression and prognostic value of AEG-1 in primary gastric cancer (GC) as well as its role in angiogenesis. The results obtained from real-time reverse transcription polymerase chain reaction and western blotting revealed the upregulation of AEG-1 mRNA (P=0.007) and protein expression (P<0.001) in the majority of cancerous tissues compared with matched adjacent non-cancerous gastric tissues. To further investigate the clinicopathological and prognostic roles of AEG-1, immunohistochemical analysis of 216 GC tissue blocks was performed. The results showed that high AEG-1 expression closely correlated with differentiation degree (P<0.001 ), T stage (P<0.001), N stage (P=0.003) and M stage (P=0.013). Consistent with the abovementioned results, AEG-1 upregulation was also found to significantly correlate with poor survival in GC patients (P<0.001). Furthermore, carcinomas with elevated AEG-1 expression demonstrated high vascular endothelial growth factor (VEGF) expression and microvessel density, which was labeled by cluster of differentiation 34. In addition, an AEG-1 siRNA assay in MGC-803 cells showed that the AEG-1 gene may promote VEGF and hypoxia-inducible factor-1α protein and mRNA expression. The results of the current study indicated that AEG-1 may serve as a valuable prognostic marker for GC and may be involved in regulating tumor angiogenesis.

## Introduction

The incidence of gastric cancer (GC) is decreasing worldwide, however, it is the third leading cause of cancer-related mortality in China and was responsible for the mortality of 320,000 patients between 2004 and 2005 (1). Although great improvements have been made in diagnostic techniques and treatments for GC, the five-year survival rate for GC remains as low as 20 to 30% (2). It is of particular concern that GC is a multifactorial and multistep disease that involves the activation of oncogenes and inactivation of tumor suppressor genes at different stages (3,4). The GC stage at diagnosis is a significant prognostic factor. However, distant and locoregional relapses frequently occur despite surgical resection and multimodality therapy. Thus, well-characterized biomarkers are necessary for the screening, diagnosis or prognostic prediction of GC.

Astrocyte elevated gene-1 (AEG-1), also termed metadherin and LYRIC, was originally identified as a human immunodeficiency virus-inducible gene in primary human fetal astrocytes (5,6). Using phage display strategy, Brown and Ruoslahti (7) established an AEG-1-mediated metastases of mouse breast cancer cells to the lungs, which demonstrated the involvement of AEG-1 in cancer. Further studies have shown that elevated AEG-1 expression is detected in subsets of malignant tumors, including esophageal squamous cell carcinoma (8), hepatocellular carcinoma (9), non-small cell lung cancer (10), neuroblastoma (11), breast cancer (12), prostate cancer (13), and renal cancer (14), compared with normal cells and matched non-neoplastic tissues. AEG-1 is vital in the biological functions of cancer by influencing invasion, metastasis (15), chemoresistance (16), autophagy (17) and tumor growth (18). In addition, Emdad *et al* (19) reported that representative angiogenic markers, including angiopoietin 1 and hypoxia-inducible factor (HIF)-1α, correlate with AEG-1 upregulation in rat embryo fibroblasts that were transduced by AEG-1. Furthermore, AEG-1-induced angiogenesis involved the activation of phosphoinositide 3-kinase/Akt signaling and the ectopic expression of AEG-1 in human umbilical vein endothelial cells promoted tube formation.

The aim of the present study was to analyze AEG-1 expression levels in GC using immunohistochemistry, western blotting and real-time reverse transcription-polymerase chain reaction (qPCR). In addition, the possible correlation between AEG-1 expression and clinicopathological variables was investigated and its prognostic value was determined. Furthermore, the functional role of AEG-1 in the angiogenesis of GC was evaluated.

## Materials and methods

### Case selection

In the present study, a total of 216 paired cancerous and matched adjacent non-cancerous gastric mucosa tissues were selected consecutively from the surgical pathology archives of the First Affiliated Hospital of Sun Yat-Sen University (Guangzhou, China) between 2004 and 2005. The previous histological diagnosis was confirmed by a pathologist. Clinicopathological variables, including age, gender, histological type and pathological stage, were collected by reviewing medical charts and pathology records. Among these patients, 80 were males and 136 were females, and the age of these patients ranged between 26 and 81 years at the time of surgery (mean age, 61.9 years). All patients had follow-up records for over five years. All cases were selected for the present study on the basis of a paraffin-embedded, formalin-fixed tissue block. Approval for this study was provided by the Medical Ethics Committee of Sun Yat-Sen University (Guangzhou, China), and all specimens were anonymous and handled according to the ethical and legal standards.

### Immunohistochemistry for AEG-1, vascular endothelial growth factor (VEGF) and cluster of differentiation (CD)34

Unstained 4-μm sections were cut from the selected paraffin block and deparaffinized by routine techniques. The slides were steamed for 20 min in sodium citrate buffer (diluted to 1X from 10X heat-induced epitope retrieval buffer). After cooling for 5 min, the slides were labeled for 2 h at room temperature with a 1:100 dilution of rabbit monoclonal antibody against AEG-1, 1:200 dilution of rabbit monoclonal antibody against VEGF or 1:200 dilution of mouse monoclonal antibody against CD34 (all Maxim-Bio, Fuzhou, China). Labeling was detected by adding biotinylated secondary antibodies, avidin-biotin complex and 3,3′-diaminobenzidine. The sections were counterstained with hematoxylin. AEG-1, VEGF and CD34 immunolabeling were evaluated jointly by two of the authors using a multi-headed microscope (Olympus Corporation, Tokyo, Japan), with agreement on all cases. In the negative control, phsophate-buffered saline was used to replace AEG-1, VEGF and CD34. The known positive slice in the streptavidin-peroxidase kit (Maxim-Bio) was used as the positive control.

### Evaluation of immunohistochemistry

The scoring criteria were determined during a preliminary evaluation using a multi-headed microscope in order to reach a consensus. The staining results for each antibody were interpreted by two of the authors independently, without prior knowledge of the clinicopathological parameters. Discordant cases were reviewed and agreed upon prior to statistical analysis of the data. For each sample, at least five fields (magnification, ×400) and >500 cells were analyzed. Under a microscope, the distribution, positive intensity and positive ratio of AEG-1 and VEGF protein expression were observed. The number of immunopositive cells was semi-quantitatively estimated. Firstly, a scoring system according to the staining intensity was determined as follows: 0, colorless; 1, light yellow; 2, brown-yellow; and 3, dark brown. Scoring according to the percentage of positive cells was determined as follows: 0, no positive cells; 1, <10% positive stained cells; 2, 11–50% positive stained cells; 3, 51–75% positive stained cells; and 4, >75% positive stained cells. If the product of multiplication between staining intensity and the percentage of positive cells was ≥2, the sample was considered to be immunopositive (+). A known positive control was included with each run of staining to monitor the batch-to-batch consistency.

### Microvessel density (MVD) counting

The previously mentioned pathologist performed the MVD scoring. MVD was determined by light microscopy in the regions of invasive tumor containing the highest numbers of capillaries and small venules (microvessels) per area (i.e. areas with the most intense neovascularization). Tumor sections were scanned first at a low power (magnifications, ×40 and ×100) to identify areas of invasive carcinoma with the greatest numbers of distinct CD34-stained microvessels per area (brown), usually at the margins of the carcinoma. Individual microvessel counts were performed on a ×200 field within the area of the most intense tumor neovascularization. Any endothelial cell or endothelial cell cluster that was positive for CD34 and clearly separate from an adjacent cluster was considered to be a single countable microvessel. Data are presented as the highest number of microvessels identified within any single ×200 field. The review was performed without any knowledge of the clinical outcome.

### Cell culture and RNA interference

The MGC-803 cell line was purchased from the Wuhan Cell Bank of Wuhan University (Wuhan, China). The cells were cultured in RPMI medium supplemented with 10% heat-inactivated fetal calf serum at 37°C under 5% CO_2_ atmosphere in a humidified incubator. Lipofectamine 2000 was used for siRNA transfections. MGC-803 cells in the exponential phase of growth were grown for 24 h, plated in antibiotic-free RPMI at a density of 2×10^4^ cells/ml and then transfected with siRNA (AEG-1 siRNA, >97% purity). The ion-exchange high-performance liquid chromatography-purified siRNA (AEG-1 siRNA) was purchased from Ruibo (Guangzhou, China). For selection of the AEG-1 siRNA, a homo sapiens AEG-1 mRNA sequence was subjected to the National Center for Biotechnology Information Basic Local Alignment Search Tool search against the *Bos taurus* expressed sequence tag cDNA library. The following base pair duplexes of siRNA were used: Sense, 5′-GGUCUCAGAUGAUGAUAAATT-3′ and antisense, 5′-UUUAUCAUCAUCUGAGACCTT-3′ for AEG-1. In addition to the medium control, the cells were transfected with negative control siRNA and following 24, 48 and 72 h of transfection, the cells were harvested and used for the experiments.

### Western blotting

Protein extracts were prepared using a lysis buffer (10 mM Tris-HCl [pH 7.5], 1% Triton X-100, 20% glycerol, 1 mM EDTA, 50 mM NaCl and 1 mM phenylmethylsulfonyl fluoride). In total, 90 μg protein was loaded onto SDS-polyacrylamide gels, subjected to electrophoresis and transferred onto nitrocellulose membranes (Millipore, Billerica, MA, USA). Blotted membranes were incubated with a 1:2,000 dilution of the anti-AEG-1, -VEGF and -HIF-1α antibodies (Sigma-Aldrich, St. Louis, MO, USA) in 5% milk/Tris-buffered saline with Tween-20 (TBST) for 24 h. Following three 10-min washes in TBST, the membranes were incubated with a 1:1,000 dilution of goat horseradish peroxidase-conjugated secondary antibody (Sigma-Aldrich) in 5% milk/phosphate-buffered saline with Tween-20 (PBST) for 3 h. Finally, membranes were subjected to three 10-min washes in PBST and the immunocomplexes were visualized using an enhanced chemiluminescence system (Amersham Pharmacia Biotech, Amersham, UK). The same membrane was reprobed with β-actin-specific antibody to ensure equal control.

### qPCR

For qPCR, total RNA was extracted using the RNA easy kit (Qiagen, Valencia, CA, USA). Briefly, total RNA (1 μg) was reverse transcribed in 20 μl reaction using 0.5 μg oligo dT and 200 units of Superscript II RT (Invitrogen Life Technologies, Carlsbad, CA, USA). The amplification was performed in a total volume of 20 μl, containing 0.5 μM of each primer, 4 mM MgCl_2_, 2 μl LightCycler™ FastStart DNA Master SYBR Green I (Roche Diagnostics, Indianapolis, IN, USA) and 2 μl of cDNA (1:10). The Ct value (initial amplification cycle) of each standard dilution was plotted against the standard cDNA copy numbers. The sample cDNA copy number was calculated according to the sample Ct value and on the basis of the standard curves for each gene. Standard curves and PCR results were analyzed using ABI 7000 software (Applied Biosystems, Foster City, CA, USA). The results were normalized against those of the housekeeping gene, glyceraldehyde-3-phosphate dehydrogenase (GAPDH), in the same sample. The target gene primers used were: Forward, 5′-CGAGAAGCCCAAACCAAATG-3′ and reverse, 5′-TGGTGGCTGCTTTGCTGTT for AEG-1; forward, 5′-CAAGGCCAGCACATAGGAGA-3′ and reverse, 5′-ACGCGAGTCTGTGTTTTTGC-3′ for VEGF; forward, 5′-AAGTCAGCAACGTGGAAGGT-3′ and reverse, 5′-TTCATATCGAGGCTGTGTCG-3′ for HIF-1α; and forward, 5′-GACTCATGACCACAGTCCATGC-3′ and reverse, 5′-AGAGGCAGGGATGATGTTCTG-3′ for GAPDH. All of the PCR reactions were performed in duplicate.

### Statistical analyses

Statistical analysis was performed using the one- or two-way analysis of variance test followed by Tukey’s test or Student’s t-test, and Spearman’s rho correlation analysis in SPSS 11.5 (SPSS, Inc., Chicago, IL, USA). The probability of survival in the different subgroups was calculated using the Kaplan-Meier method and statistical significance was analyzed using the log-rank test. P<0.05 was considered to indicate a statistically significant difference.

## Results

### Analysis of AEG-1 mRNA expression by qPCR and protein expression by western blotting

**Figure 1:**
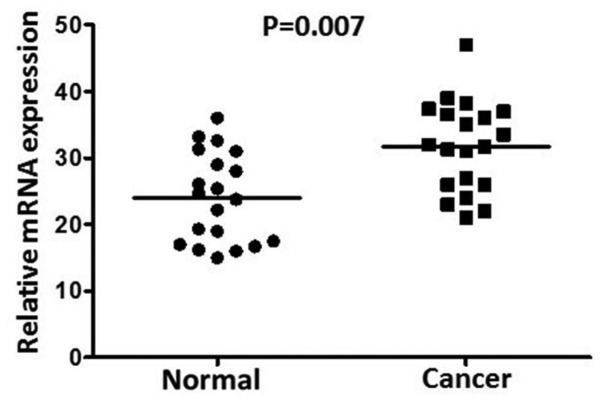
Elevated mRNA expression of astrocyte elevated gene-1 in normal gastric mucosa and gastric cancer tissues as assessed by real-time reverse transcription polymerase chain reaction (n=20; P=0.007). Horizontal lines represent the mean and data are presented as three individually matched pairs of normal and neoplastic tissue samples.

**Figure 2:**
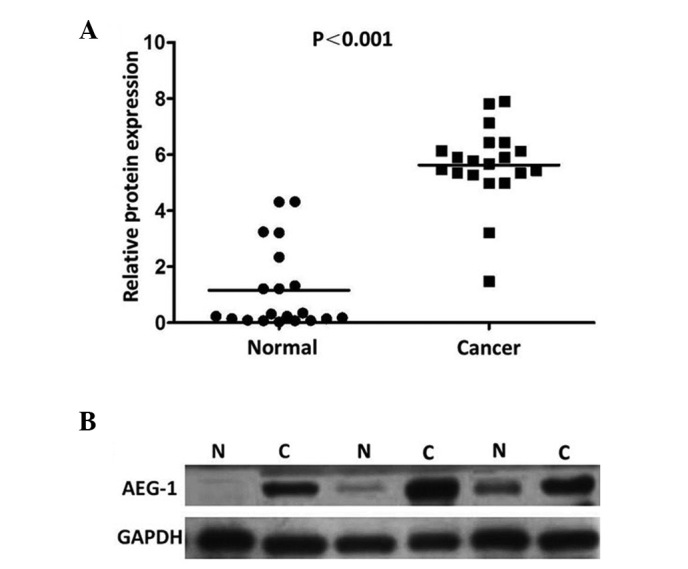
Increased protein expression of AEG-1 in GC as assessed by western blotting. (A) Relative AEG-1 protein expression levels in GC and non-cancerous tissues (AEG-1/GAPDH; n=20; P<0.001). Horizontal lines represents the mean. (B) Representative results of AEG-1 protein expression in three pairs of gastric tumor tissues and matched adjacent non-tumorous tissues. Lane N, matched non-cancerous gastric mucosa; Lane C, GC tissues; AEG-1, astrocyte elevated gene-1; GC, gastric cancer; GAPDH, glyceraldehyde-3-phosphate dehydrogenase.

To determine whether AEG-1 expression is associated with the progression of GC, qPCR and western blotting were performed on the 20 pairs of primary GC tissues and matched adjacent non-cancerous gastric mucosa tissues. The AEG-1 expression levels in the tumor-bearing tissues were significantly higher than those in the adjacent non-tumor tissues (P=0.007; Fig. 1). The results showed an AEG-1 band with a predicted size of 82 kDa and the relative amount of AEG-1 protein was measured further via densitometry. Consistent with the qPCR results, an increase in AEG-1 protein expression was observed in 19 (95.0%) of the gastric tumor tissues, compared with the matched adjacent non-tumor tissues (P<0.001; Fig. 2).

### AEG-1 expression in GC and its correlation with clinicopathological features

**Figure 3:**
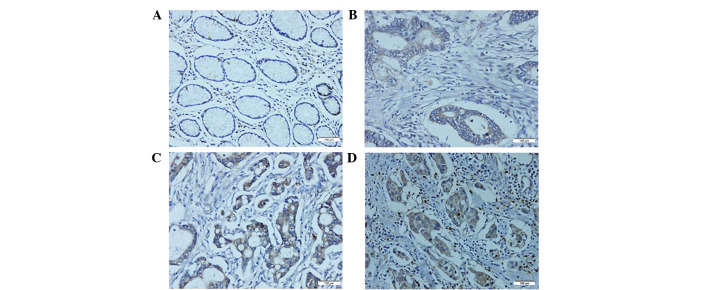
Astrocyte elevated gene-1 (AEG-1) protein expression in gastric cancer (GC) surgical specimens shown by immunohistochemistry. Immunohistochemical staining for AEG-1 was predominantly observed in the cytoplasm of the GC cells. Weak AEG-1 staining was observed in (A) the non-cancerous gastric mucosa and (B) the well-differentiated GC cells. Strong AEG-1 staining was observed in the (C) moderately and (D) poorly differentiated GC cells (stain, 3,3′-diaminobenzidine; scale bar, 100 μm; magnification, ×200).

To further investigate the clinicopathological and prognostic roles of AEG-1 expression, immunohistochemical analyses was performed on the 216 paraffin-embedded GC tissue blocks. In total, 143 of the 216 (66.2%) cases showed high AEG-1 expression in cancerous tissues, whereas 34 of the 216 (15.7%) cases showed high AEG-1 expression in the normal gastric mucosa (Fig. 3). The correlation between the expression of AEG-1 and various clinicopathological parameters are listed in Table I. The results indicated that increased expression of AEG-1 was significantly correlated with the differentiation degree (P<0.001), depth of tumor infiltration (T stage; P<0.001), the N stage (P=0.003) and the M stage (P=0.013), whereas AEG-1 was not found to correlate with age, gender, tumor size or histological type.

### Correlation between AEG-1 expression and GC survival

**Figure 4:**
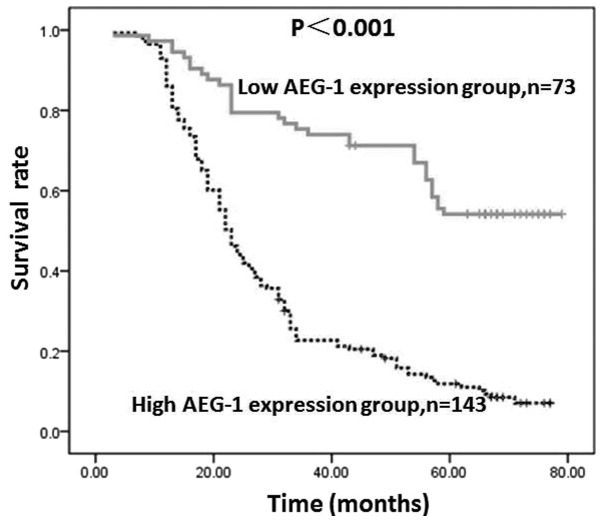
Kaplan-Meier survival curves of gastric cancer patients (n=216). The survival rate of patients in the high astrocyte elevated gene-1 (AEG-1) expression group was significantly lower than that of patients in the low AEG-1 expression group (log-rank test; P<0.001).

AEG-1 expression by GC cells in tumor lesions was found to inversely correlate with patient survival, which was revealed by Kaplan-Meier analysis and the log-rank test. The five-year overall survival rates in patients with high and low AEG-1 expression were 9.8 and 50.7%, respectively. As shown in Fig. 4, the two survival curves were significantly different and the survival rate in the group with low AEG-1 expression was higher than that in the group with high AEG-1 expression (P<0.001).

### Correlation between AEG-1, and VEGF and MVD

**Figure 5:**
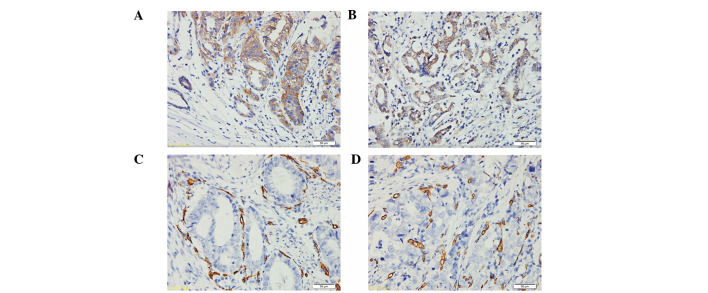
Immunohistochemical staining for vascular endothelial growth factor (VEGF) is predominantly observed in the cytoplasm of lung cancer cells. (A) Strong positive expression of VEGF was observed in the moderately differentiated adenocarcinoma. (B) Positive expression of VEGF was observed in the moderately differentiated squamous carcinoma and CD34 staining was predominantly located in the surrounding microvessels of the lung carcinoma. Positive expression of CD34 was observed in the moderately differentiated (C) adenocarcinoma and (D) squamous carcinoma (streptavidin-peroxidase; scale bar, 50 μm; magnification, ×200).

AEG-1 and VEGF protein expression was examined in the 216 cases of primary GC samples. The results revealed a positive correlation between AEG-1 and VEGF protein expression in the GC samples (P<0.001). VEGF was expressed in the cytoplasm of tumor cells with a homogenous or granular pattern (Fig. 5A and B). In all GC patients, 111 of the 143 cases with high AEG-1 expression showed VEGF positivity (77.6%). Of the 73 cases with reduced AEG-1, nine cases were also found to exhibit VEGF positivity (12.3%). CD34-positive granules were located in the vascular endothelial cells (Fig. 5C and D) and MVD in the AEG-1-positive group was 78.06±6.79, which was markedly higher than that in the AEG-1-negative group (17.72±3.31). AEG-1 staining was found to positively correlate with MVD (P<0.001; Table II).

### AEG-1 siRNA inhibits the expression of AEG-1 in MGC-803 cells

**Figure 6:**
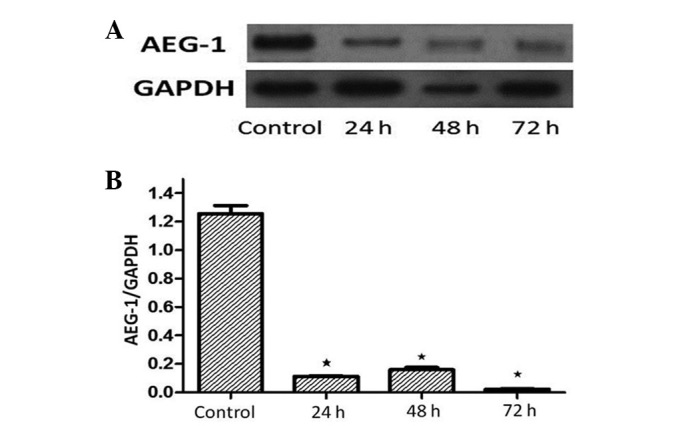
Representative western blotting for AEG-1 protein expression shows downregulation of AEG-1 by siRNA. Data are presented as the means ± standard deviation of three independent experiments. ^*^P<0.05 vs. scramble siRNA (control). AEG-1, astrocyte elevated gene-1; GAPDH, glyceraldehyde-3-phosphate dehydrogenase.

To investigate the role of AEG-1 on VEGF and HIF-1α expression in MGC-803 cells, siRNA was used to specifically knockdown AEG-1 expression. The efficacy of AEG-1 siRNA on the AEG-1 protein was confirmed by western blotting at 24, 48 and 72 h following siRNA transfection. As shown in Fig. 6, treatment with AEG-1 siRNA significantly decreased AEG-1 protein expression by ~80% in the MGC-803 cells at 48 and 72 h when compared with the siRNA control (P<0.01). This indicated that the AEG-1 siRNA achieved a successful knockdown.

### Role of AEG-1 in VEGF, and HIF-1α mRNA and protein expression in MGC-803 cells

**Figure 7:**
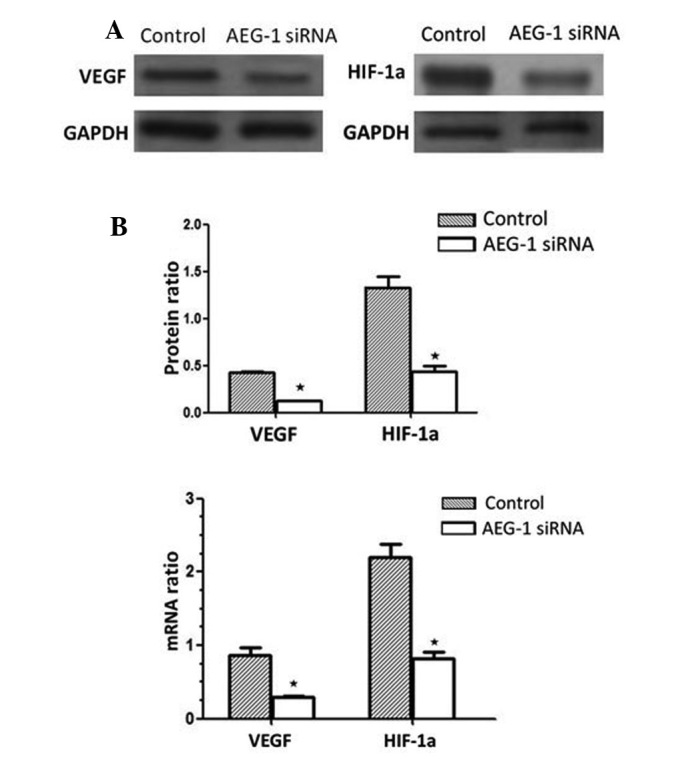
Effects of AEG-1 siRNA on VEGF and HIF-1α expression. MGC-803 cells were transfected with AEG-1 siRNA and Con-siRNA. (A) VEGF and HIF-1α were determined by western blotting. Upper panel shows a representative blot for VEGF and HIF-1α protein, and the lower panel shows the quantification of VEGF and HIF-1α protein levels. (B) VEGF and HIF-1α mRNA were determined by real-time reverse transcription polymerase chain reaction in MGC-803 cells and the levels of VEGF and HIF-1α mRNA are presented as the (VEGF and thrombospondin-1)/GAPDH mRNA ratio. Data are presented as means ± standard deviation of three independent experiments. ^*^P<0.05 vs. Con-siRNA (control). AEG-1, astrocyte elevated gene-1; VEGF, vascular endothelial growth factor; HIF-1α, hypoxia-inducible factor-1α; Con-siRNA, scramble siRNA; GAPDH, glyceraldehyde-3-phosphate dehydrogenase.

In response to AEG-1 siRNA, VEGF protein expression decreased by 67.23% following AEG-1 siRNA transfection, compared with the siRNA-transfected control group (P<0.01). Furthermore, HIF-1α protein expression decreased by 69.37% compared with the siRNA-transfected control group (P<0.01; Fig. 7A). These results were associated with a significant decrease in VEGF and HIF-1α mRNA expression in AEG-1 siRNA-transfected MGC-803 cells (Fig. 7B), and indicated that AEG-1 signaling induces VEGF and HIF-1α upregulation in MGC-803 cells.

## Discussion

GC is one of the most frequently diagnosed malignant neoplasms and has a poor prognosis despite curative surgery and postoperative adjuvant therapy (20). It has long been known that the tumorigenesis and progression of GC is the result of a combination of environmental factors, and the accumulation of generalized and specific genetic alterations. A number of genetic or epigenetic alterations have previously been reported in GC, including loss of heterozygosity, microsatellite and chromosomal instability, as well as hypermethylation (21). Due to the early metastasis and marked invasion, the identification of GC-specific biomarkers, which are involved in these processes is significanct for the diagnosis, therapy and prognostic prediction of GC in clinics.

Previously, AEG-1 overexpression has been found in a spectrum of cancer types, including breast cancer, glioma and prostate cancer. Furthermore, elevation of AEG-1 expression has been found to markedly correlate with the clinical characteristics of these tumors (12,22,23). In addition, high expression of AEG-1 has been demonstrated to promote cell proliferation, cell transformation and tumor progression (24). The previously described reports indicated that AEG-1 may be closely involved in promoting tumorigenesis or progression. However, thus far, few studies have analyzed the expression and clinical significance of AEG-1 in primary GC. Therefore, the present study detected AEG-1 expression in GC by qPCR, western blotting and immunohistochemistry, and analyzed the clinicopathological and prognostic significance of AEG-1 in a large number of patient samples.

AEG-1 mRNA expression was investigated by qPCR, and protein expression was investigated by western blotting detection in 20 pairs of primary GC tissues and matched adjacent non-cancerous gastric mucosa tissues. The results showed that the AEG-1 mRNA and protein levels were significantly upregulated in the tumor tissue samples, compared with the levels observed in the adjacent non-tumor tissue samples, which is consistent with the observations made by Gnosa *et al* (25). In addition, the immunohistochemical results demonstrated high AEG-1 expression in 66.2% (143/216) of GC patients, which was significantly higher than that identified in the adjacent non-tumor tissue samples. These results were consistent with an earlier hypothesis by Lee *et al* (24) that AEG-1 may be an oncogene; furthermore, it was hypothesized that AEG-1 activation may be important in the tumorigenesis or progression of GC.

Additionally, activation of the nuclear factor-κB signal by AEG-1 may be a key molecular mechanism by which AEG-1 promotes anchorage-independent growth and invasion, two typical features of the neoplastic phenotype (26). In the current relatively large series of GC patients (n=216), high AEG-1 expression significantly correlated with a higher T stage of GC, implying that AEG-1 regulates tumor growth and invasion. Further analysis concerning the correlation between AEG-1 expression and clinical characteristics also showed a significant correlation the with N and M classifications, although, AEG-1 expression was not found to correlate with the age, gender, tumor size and histological type. This indicated that AEG-1 may be useful as an independent marker to identify subsets of GC patients with greater certainty. In addition, enhanced AEG-1 immunoreactivity was detected in the poorly differentiated GC tissues compared with the normal gastric tissues and the well-differentiated GC tissues, which suggested that high AEG-1 expression may be involved in tumor progression. These results were consistent with the observation made by Li *et al* (12), describing a correlation between high AEG-1 expression and breast carcinomas. The results obtained from the Kaplan-Meier survival analysis of the current study showed that patients with high AEG-1 expression exhibited significantly shorter overall survival times than patients with low AEG-1 expression. These results indicated that COP1 may serve as a valuable prognostic biomarker for GC patients following surgery and as a potential target for gene therapy in the treatment of GC.

The malignant potential of cancer involves multifactor and multistep processes that occur in a specific manner during tumor progression (27). This ‘angiogenic switch’, as it is termed, is necessary for tumors to obtain the required nutrients and oxygen to grow, and its importance in the growth of solid tumors has been well established (28). New vessels, that are produced by the primary tumor and secondary distant metastases, reflect a net balance between positive and negative regulators of angiogenesis (29). The abovementioned observations emphasize that any genetic change in a cancer cell that culminates in tumor growth and metastasis are likely to be inexorably bound to angiogenesis. The results of the current study also showed that MVD in the AEG-1-positive group was 78.06±6.79, which was markedly higher than that observed in the AEG-1-negative group (17.72±3.31; P<0.001). In addition, the GC cases with high AEG-1 expression exhibited high levels of additional angiogenic markers, including VEGF, which indicated that AEG-1 may promote angiogenesis and be important in tumor angiogenesis. HIF-1 is expressed in hypoxic tumor cells and activates various hypoxia-responsive genes, which enhance tumor growth, invasion and metastasis (30). To elucidate the detailed molecular mechanism underlying AEG-1 function as an angiogenesis promoter, the focus of the present study was on the expression of HIF-1α and VEGF in MGC-803 cells that were treated with AEG-1 siRNA. In these contexts, AEG-1 was shown to enhance HIF-1α expression in MGC-803 cells. In addition, HIF-1 activates proangiogenic cytokines, such as VEGF, which increase the regrowth of tumor blood vessels (31). In the present study, AEG-1 was found to upregulate VEGF expression in MGC-803 cells, which is consistent with the observations of Yoo *et al* (9). In addition, the results further demonstrated that HIF-1α and VEGF may be vital downstream genes of AEG-1, which are important in angiogenesis that is mediated by AEG-1. However, the observation of an ~70–80% reduction of HIF-1α and VEGF expression in MGC-803 cells treated with AEG-1 siRNA indicated that other signaling molecules may partially contribute to increased hypoxia-induced angiogenesis. Further studies are required to clarify the complex mechanisms involved in GC angiogenesis.

In conclusion, the results of the present study, which are based on immunohistochemical and molecular genetic methods, indicate a frequent and complex role of AEG-1 in the pathogenesis of GC. Furthermore, the results indicate that AEG-1 is involved in the complex regulatory mechanism of angiogenesis, potentially by the upregulation of HIF-1α and VEGF expression.

## Figures and Tables

**Table I:** Correlation between AEG-1 expression and clinicopathological variables of 216 GC cases.

	AEG-1 expression		χ^2^ test
		
Characteristic	High, n	Low, n	P-value
Normal gastric mucosa	34	182	<0.001
GC tissues	143	73	
Age, years			0.687
≤50	49	34	
>50	94	39	
Gender			0.101
Male	47	33	
Female	96	40	
Tumor size, cm			0.401
<3	17	12	
≥3	126	61	
Histological type			0.191
Intestinal	71	27	
Diffuse	51	31	
Mixed	21	15	
Differentiation degree			<0.001
Well to moderate	31	47	
Poor	97	24	
Other	15	2	
T classification			<0.001
T1	6	27	
T2	9	24	
T3	14	12	
T4	114	10	
N classification			0.003
N0	41	34	
N1	27	17	
N2	28	13	
N3	47	9	
M classification			0.013
M0	112	67	
M1	31	6	

AEG-1, astrocyte elevated gene-1; GC, gastric cancer.

**Table II:** Correlation between AEG-1, and VEGF and MVD.

		VEGF^a^		
			
AEG-1	n	+ (n)	− (n)	MVD^a^ (mean ± SD)
+	143	111	32	78.06±6.79
−	73	9	64	17.72±3.31

a P<0.001.

AEG-1, astrocyte elevated gene-1; VEGF, vascular endothelial growth factor; MVD, microvessel density; SD, standard deviation.
